# Meta-analysis and systematic review: burosumab as a promising treatment for children with X-linked hypophosphatemia

**DOI:** 10.3389/fendo.2024.1414509

**Published:** 2024-08-15

**Authors:** Kangning Wang, Runze Zhang, Ziyi Chen, Yi Bai, Qing He

**Affiliations:** State Key Laboratory of Oral & Maxillofacial Reconstruction and Regeneration, Key Laboratory of Oral Biomedicine Ministry of Education, Hubei Key Laboratory of Stomatology, School & Hospital of Stomatology, Wuhan University, Wuhan, China

**Keywords:** X-linked hypophosphatemia, burosumab, pediatric, meta-analysis, therapeutic effectiveness

## Abstract

**Objective:**

The aim of this study was to evaluate the effectiveness of burosumab therapy in children with X-Linked Hypophosphatemia (XLH).

**Materials and methods:**

We systematically reviewed literature from PubMed, Web of Science, The Cochrane Library, and Embase up until January 2024, using EndNote Web for study organization. The Newcastle–Ottawa scale guided quality assessment, while Revman software was used for data analysis and visualization. Study selection, quality evaluation, and data aggregation were independently performed by three researchers.

**Results:**

The meta-analysis encompassed ten studies, including eight cohort studies that examined burosumab’s impact pre- and post-administration, and two randomized controlled trials comparing burosumab to standard therapy. The evidence from this review suggests burosumab’s superiority in managing XLH in pediatric populations, particularly in improving key biochemical markers including 1,25-dihydroxyvitamin D (1,25-(OH)_2_D), phosphorus, and alkaline phosphatase (ALP), alongside improvements in the renal tubular maximum reabsorption rate of phosphate to glomerular filtration rate (TmP/GFR), and significant skeletal improvements as indicated by the rickets severity score (RSS) and the 6-minute walk test (6MWT). However, the long-term safety and effects, including height and quality of life (QOL) data, remains to be elucidated.

**Conclusions:**

Burosumab has shown significant therapeutic effectiveness in treating children with XLH, highlighting its potential as a key treatment option.

## 1 Introduction

X-linked hypophosphatemia (XLH) is a rare genetic disorder primarily caused by loss-of-function variants of phosphate-regulating endopeptidase gene on the X chromosome (PHEX) located on the X chromosome. It is the most common form of hereditary rickets and osteomalacia (1, 2), while other genetic factors can also contribute to hypophosphatemic rickets, including mutations in FGF23, DMP1, ENPP1, and FAM20C (3). With a prevalence of approximately 1 in 20,000 newborns, XLH presents significant clinical challenges (4, 5). The disorder’s primary manifestations in infancy include rickets and growth retardation, which evolve into more pronounced lower limb deformities during childhood and adolescence (6). Additionally, studies indicate that XLH children also suffer from bone pain, gait impairment, reduced range of motion of ankle/knee joint, tooth abscesses, and/or skull stenosis (6–8).

XLH is characterized by reduced renal phosphate reabsorption, which can be reflected by TmP/GFR value (9, 10), and impaired production of 1,25-dihydroxyvitamin D (1,25-(OH)_2_D), complicating its management (11–13). The conventional therapy of XLH is treating the patients with oral phosphate and active vitamin D to compensate for renal phosphate wasting and counter 1,25-(OH)_2_D deficiency (14). With traditional treatment methods, the serum alkaline phosphatase (ALP) level can be successfully regulated to remain within the upper limit of normal values within a year (14). Additionally, this treatment has shown efficacy in improving bone deformities in 30% to 60% of patients, enhancing their growth velocity, and optimizing dentin mineralization (14–16). While this approach can modestly improve bone deformities and growth rates in pediatric patients, its efficacy varies, and long-term use is associated with significant adverse events, including hyperparathyroidism, hypercalciuria, renal calcinosis, and kidney stones (14, 17). Despite treatment, hypophosphatemia often remains unresolved, and full restoration of muscle function is not achieved. Due to insufficient response to medication, some children require corrective surgery on their lower limbs (18). Furthermore, the frequent dosing required for conventional treatment poses a significant hurdle in ensuring patient compliance, as phosphate must be taken several times a day. These challenges underscore the urgent need for more effective and safer treatment options to enhance the quality of life for children with XLH.

In exploring alternative treatments, recent research has focused on the role of Fibroblast Growth Factor 23 (FGF23) in XLH pathophysiology. FGF23 are significantly elevated in XLH patients, which is responsible for renal phosphate wasting and suppressed 1,25-(OH)_2_D expression (19–21). Produced mainly by osteocytes and osteoblasts, FGF23 plays a crucial role in phosphate homeostasis. Its function on phosphate metabolism was initially recognized by missense variants in FGF23 discovered from children with autosomal dominant hypophosphataemic rickets (ADHR) (22). The variants found in ADHR patients prevent the normal cleavage of the intact, bioactive form of FGF23, leading to low serum phosphate concentrations and rickets/osteomalacia (11, 23). FGF23 regulates the reabsorption of phosphorus by inhibiting the expression of the sodium phosphate co-transporters, NPT2a and NPT2c, at the renal proximal tubules (24). It also represses the synthesis of active 1,25-(OH)_2_D and enhances its degradation by upregulating the renal 24-hydroxylase enzyme (CYP24A1) and downregulating the 1α-hydroxylase enzyme (CYP27B1) (25, 26). Moreover, FGF23 impacts parathyroid hormone (PTH) expression, influencing blood calcium and phosphate metabolism and ultimately affecting bone mineralization, leading to compensatory changes in osteoblast activity and alkaline phosphatase secretion (27).

Burosumab, a monoclonal antibody targeting FGF23, has emerged as a promising new treatment. Approved by the European Medicines Agency, the U.S. Food and Drug Administration, and other regulatory bodies for both pediatric and adult XLH patients, burosumab works by blocking FGF23, thereby increasing renal phosphate reabsorption and enhancing serum levels of phosphorus and active vitamin D. Clinical trials have demonstrated that burosumab significantly improves serum phosphate levels, increases active vitamin D levels, and enhances renal phosphate reabsorption. Additionally, the therapeutic effects of burosumab extend beyond biochemical improvements. QOL assessments have shown marked enhancements in patients with burosumab treatment, with reports of reduced pain, increased physical activity, and overall better well-being (28, 29). There is evidence of sexual dimorphism in XLH severity, with males often exhibiting more severe symptoms than females (5, 30). Burosumab’s effectiveness appears consistent across genders, though further studies are needed to confirm this (31, 32). Furthermore, burosumab has a favorable safety profile with fewer adverse events compared to conventional therapy, making it a more viable long-term treatment option (33). These benefits underscore burosumab’s potential to offer a more comprehensive and effective treatment solution for XLH, addressing both the physiological and QOL aspects of the disorder. However, the specific impact of burosumab on children with XLH warrants careful evaluation through clinical research.

This article aims to conduct a comprehensive meta-analysis and systematic review of the available clinical trials on burosumab’s use in treating pediatric XLH. We intend to scrutinize multiple study data, critically evaluate the advantages and limitations of burosumab, and offer a reliable assessment for future drug research and development directions. Our goal is to contribute to the growing body of knowledge on XLH treatment and to provide insights that may guide clinical practice and improve patient outcomes.

## 2 Materials and methods

### 2.1 Protocol and registration

This study was conducted and reported in accordance with the Preferred Reporting Items for Systematic Review and Meta-Analysis (PRISMA) checklist (34). It has been registered in the Prospero database with the registration ID CRD42023424461.

### 2.2 Eligibility criteria

Articles evaluating the therapeutic impact of burosumab in pediatric XLH patients, in comparison to other treatment modalities, were considered. There were no restrictions on publication date or language. The inclusion criteria were structured according to the PICOS question as follows:

Population (P): Pediatric individuals diagnosed with XLH.

Intervention (I): Burosumab treatment.

Comparison (C): Other treatment approaches.

Outcome (O): Rickets severity and related parameters.

Study design (S): Cross-sectional, randomized-control, and cohort studies examining serum parameters, rickets development, or walking ability among participants.

Exclusion criteria encompassed studies without a control group of pediatric XLH patients not receiving burosumab, those not reporting relevant serum parameters, rickets development, or walking ability, case reports or series, literature reviews, studies lacking statistical analysis, and qualitative studies. Studies focusing on outcomes other than rickets were also excluded.

### 2.3 Information sources

Our search encompassed four electronic databases: PubMed (https://www.pubmed.gov), Web of Science (https://www.isiknowledge.com), The Cochrane Library (https://www.cochranelibrary.com), and Embase (https://www.embase.com) from their inception up to January 2024. The list of identified studies was organized using EndNote X9, and duplicate records were removed.

### 2.4 Search strategy

The search strategy included terms: ((Burosumab or KRN23 or Crysvita) AND (X-linked hypophosphataemia or XLH or hypophosphataemic rickets) AND (pediatric OR children)).

### 2.5 Study selection

An initial pool of 667 articles was identified, which was subsequently narrowed down to ten publications for inclusion in the meta-analysis. This selection process, conducted by reviewers Kangning Wang, Runze Zhang, and Ziyi Chen, involved three stages: first, the use of EndNote X9 to identify and eliminate duplicate records; second, an individual review of titles and abstracts for relevance; and third, a full-text analysis to finalize selections. Discrepancies were resolved through consensus.

### 2.6 Data collection process and data items

Three reviewers (Kangning Wang, Runze Zhang, and Ziyi Chen) independently extracted data from the selected articles, including study design, timeframe, follow-up period, participant demographics (country, setting, age, sex distribution), interventions, and outcomes (serum phosphorus levels, serum 1,25-(OH)_2_D levels, ALP levels, TmP/GFR, rickets severity score (RSS), Height Z-Score, 6-minute walking test results (6MWT), etc.). Authors of the studies were contacted for additional information when necessary.

### 2.7 Risk of bias in individual studies

The risk of bias was independently evaluated by the three reviewers (Kangning Wang, Runze Zhang, and Ziyi Chen) using the Newcastle–Ottawa scale for randomized-control and cross-sectional studies. Each item in the selection and exposure groups was eligible for a maximum score of one point, while each item in the comparability group could receive a maximum of two points. The highest possible score was nine. Studies were scored and categorized into high (7–9), medium (4–6), or low quality (below 3).

### 2.8 Data synthesis and statistical analysis

Estimation of aggregate effect size and forest plot generation were performed with the RevMan 5.4 software. In experiments with a before-and-after control of the cohort studies, a cohort analysis was performed, and randomized controlled trials were analyzed using randomized-control study methodology. The confidence interval (CI) for the included studies in the forest plot was set at 95%. The standardized mean differences (SMDs) with the 95% CIs of each parameter were calculated, and the significance threshold was set at P < 0.05. Subgroup analysis evaluated the effects between burosumab treatment and conventional treatment, calculating combined effect size (ES) and variance, and displayed using forest plots. The I² (percentage of variability in the effect sizes), tau-squared (between-study variance), and Cochran’s Q test (difference between the observed effect sizes and the fixed-effect model estimate of the effect size) statistics were tested for statistical heterogeneity.

## 3 Results

### 3.1 Study selection

**Figure 1:**
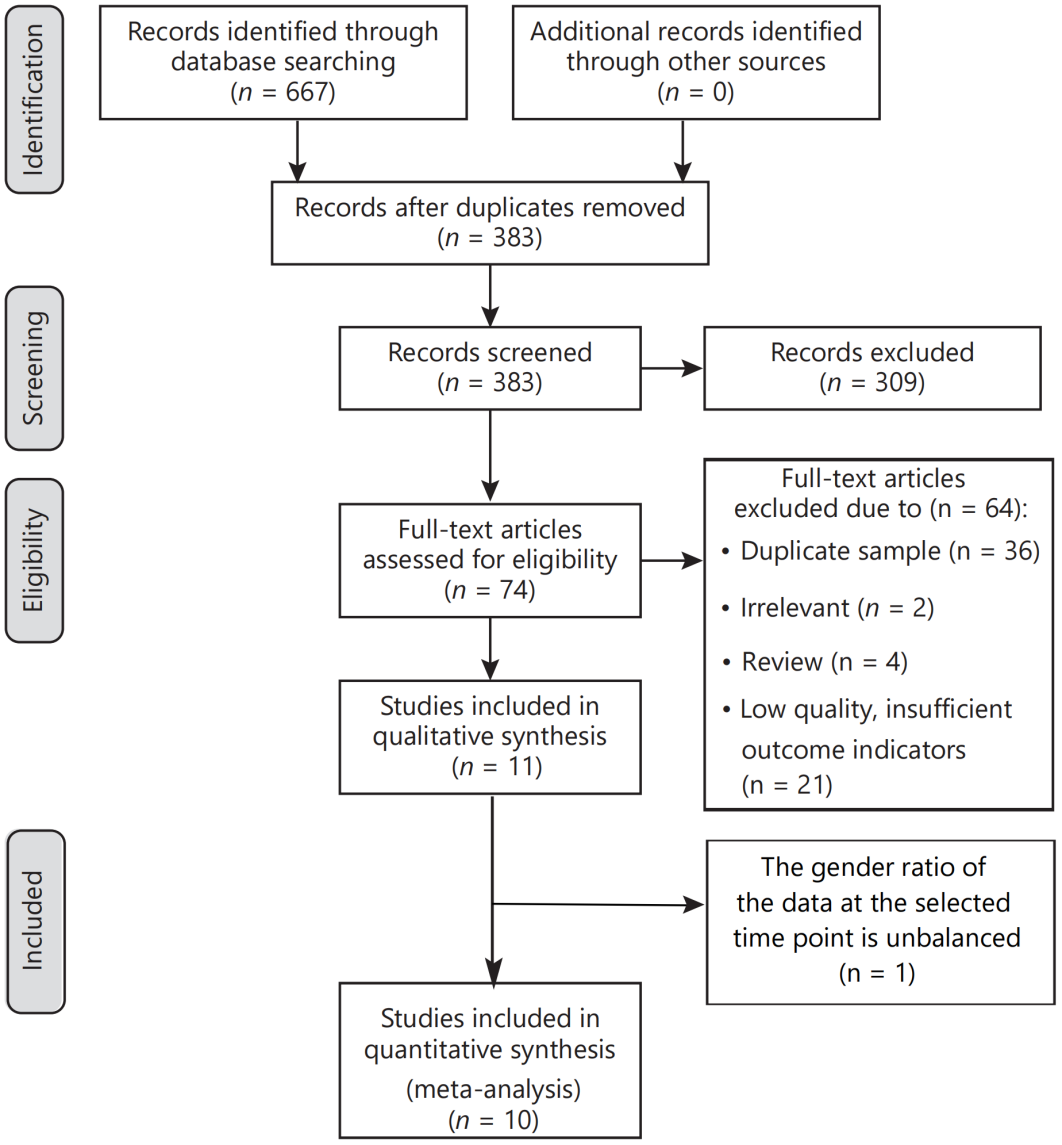
Flow diagram of the systematic review and meta‐analysis.

Our electronic searches yielded 667 titles and abstracts. In the initial screening phase, 383 titles and abstracts were reviewed, with 284 excluded due to duplication. Subsequently, 74 studies underwent full-text analysis. Reasons for exclusion at this stage included experimental duplication (36 articles), irrelevance to the topic (2 articles), reviews (4), and insufficient outcome indications (21 documents). Of the 11 studies initially included in qualitative synthesis, one was excluded due to an unbalanced gender ratio at the selected time point. Consequently, ten publications were included in this systematic review and meta-analysis. Six of these compared burosumab with conventional therapy in children (31, 32, 35–38), while the other four assessed burosumab’s efficacy and safety versus control (39–42). A thorough manual search did not yield additional articles. The article selection process is visually depicted in **Figure 1**.

### 3.2 Characteristics of the included articles

**Table 1** summarizes key characteristics of the included studies, covering ten distinct experiments on burosumab’s effectiveness in XLH patients. One study examined the efficacy of burosumab administered biweekly (Q2W) versus every four weeks (Q4W), transitioning to Q2W after 64 weeks (41). One study was controlled trials comparing burosumab with an active control group (32). Two trials explored the effects of biweekly burosumab injections over periods ranging from 114.8 weeks to 160 weeks, involving participants aged 2.94 ± 1.146 to 7.40 ± 3.40 years on average (39, 42). Six studies investigated the transition from conventional therapy to Q2W burosumab injections (31, 35–38, 40), with one study noting that 94% of participants had previously received conventional therapy, and this trail was not included in subgroup analysis (40). The studies adopted the age of twelve as the demarcation point to differentiate between children and adolescents. Six studies exclusively included children (32, 37–39, 41, 42), while four studies included both children and adolescents (31, 35, 36, 40). In one of these studies, treatment outcomes for children and adolescents were documented separately, and for our analysis, we solely incorporated the results pertaining to children (31). Sample sizes across these studies varied from 5 to 93 participants, with diverse injection dosages, cycles, and experimental durations. At the final time point chosen (40 weeks), among the studies that measured TmP/GFR, six studies included the TmP/GFR of all patients (31, 36–40). Due to the difficulty of collecting urine samples from young children, the studies by Imel et al. and Linglart et al. did not measure the TmP/GFR of all participants (32, 41). Specifically, the study by Imel et al. omitted 10 participants (6 from the burosumab group and 4 from the control group), and the study by Linglart et al. omitted 3 participants (2 from the burosumab group and 1 from the control group).

**Table 1:** Characteristics of included studies.

Imel, E. A. et al. (2019) (32)							
Methods				Study design: randomized control study Time frame: 2016 to 2019 Follow-up period: 140 weeks Excluded: 61			
Participants				Country: International Setting: National multicentre study Children with XLH, serum phosphorus <3.0 mg/dL (<0.97 mmol/L) Number: 61 Mean age: 6.27 ± 3.307 (1 to 12) Sex(M/F): 27/34			
Interventions				Active control(32) Multiple daily doses of oral phosphate and one or more daily doses of active vitamin D therapy, titrated and individualized by the investigator based on published recommendations during the Treatment Period (up to Week 64) During the Treatment Extension Period (Week 64 to Week 140), participants crossed over to receive a starting dose of SC burosumab 0.8 mg/kg Q2W Burosumab(29) 0.8 mg/kg starting dose, administered Q2W by SC injection during the Treatment Period (up to Week 64) During the Treatment Extension Period (Week 64 to Week 140), participants continued to receive a starting dose of SC burosumab 0.8 mg/kg Q2W			
Outcomes				RGI-C RSS Total Score RGI-C Long Leg Score Height-For-Age Z-Scores Growth Velocity Z Score Serum Phosphorus 1,25(OH)_2_D TmP/GFR Serum ALP PROMIS Pediatric Pain Interference, Physical Function Mobility and Fatigue Domain Scores FPS-R 6MWT Total Distance			
Notes				Participants in Japan and Korea did not enter the Treatment Extension Period Primary outcome was RGI-C Global Score at Week 40			
Whyte, M. P. et al. (2018) (42)							
Methods				Study design: cohort study Time frame: 2016 to 2019 Follow-up period: 160 weeks Excluded: 13			
Participants				Country: USA Setting: University teaching hospital Children with XLH, Serum fibroblast growth factor 23 (FGF23) level > 30 pg/mL, Serum phosphorus < 3.0 mg/dL (0.97 mmol/L), Serum creatinine within age-adjusted normal range Number: 13 Mean age: 2.94 ± 1.146 (1 to 4) Sex(M/F): 9/4			
Interventions				Burosumab subcutaneous (SC) injections every 2 weeks (Q2W) for a total of 160 weeks			
Outcomes				Serum Phosphorus Number of Participants With Adverse Events (AEs), Treatment Emergent AEs (TEAEs), Serious TEAEs, and TEAEs Leading to Discontinuation RGI-C Score RSS Total Score RGI-C Lower Limb Deformity Score Recumbent Length/Standing Height Serum Alkaline Phosphatase			
Notes				Primary outcomes were Change From Baseline at Week 40 in Serum Phosphorus and Number of Participants With Adverse Events (AEs), Treatment Emergent AEs (TEAEs), Serious TEAEs, and TEAEs Leading to Discontinuation One participant was withdrawn by subject			
Linglart, A. et al. (2019) (41)							
Methods				Study design: cohort study Time frame: 2014 to 2018 Follow-up period: 160 weeks Excluded: 52			
Participants				Country: USA Setting: University teaching hospital Children with XLH, Serum phosphorus ≤ 2.8 mg/dL (0.904 mmol/L), Serum creatinine within age-adjusted normal range Number: 52 Mean age: 8.5 ± 1.87 (5 to 12) Sex(M/F): 24/28			
Interventions				Burosumab Q2W(26) Burosumab SC injections every 2 weeks (Q2W). Dose is determined by the participant’s weight and prescribed dose by their study doctor. Burosumab Q4W Then Q2W Burosumab subcutaneous (SC) injections every 4 weeks (Q4W). Dose is determined by the participant’s weight and prescribed dose by their study doctor. Participants in Q4W were to switch to Q2W beginning with Week 64 dosing.			
Outcomes				RSS Total Score Serum Phosphorus Serum 1,25(OH)_2_D TmP/GFR RSS Knee Scores RSS Wrist Scores RGI-C Global Scores RGI-C Knee Scores RGI-C Wrist Scores Growth Velocity Growth (Standing Height, Sitting Height, Arm Length, Leg Length) 6MWT Distance POSNA-PODCI (Normative Score) Upper Extremity Scale Scores POSNA-PODCI (Normative Score) Transfer and Basic Mobility Scale Scores POSNA-PODCI (Normative Score) Sports/Physical Functioning Scale Scores POSNA-PODCI (Normative Score) Pain/Comfort Scale Scores POSNA-PODCI (Normative Score) Happiness Scale Scores POSNA-PODCI (Normative Score) Global Functioning Scale Scores FEP P1NP CTx ALP BALP Serum Pre-Dose Concentrations of burosumab Number of Participants With Treatment Emergent Adverse Events (TEAEs), Serious Adverse Events (SAEs) and Discontinuations Due to Adverse Events (AEs)			
Notes				Primary outcome measures were: [Time Frame: Baseline, Week 40, 64, 160] Change From Baseline in RSS Total Score Over Time Change From Baseline in Serum Phosphorus Over Time Change From Baseline in Serum 1,25(OH)_2_D Over Time Change From Baseline in TmP/GFR Over Time			
Namba, N. et al. (2022) (37)							
Methods				Study design: cohort study Time frame: 2017 to 2022 Follow-up period: 121.7 weeks Excluded: 15			
Participants				Country: Japan Setting: Hospital Children aged ≥ 1 and ≤12 years, Patients who have open growth plate, Willing to perform a self-administration of KRN23 and available to perform a self-administration, Diagnosis of XLH Number: 15 Mean age: 6.70 ± 3.20 (1 to 12) Sex(M/F): 2/13			
Interventions				Burosumab start with 0.8 mg/kg, and adjusted based on serum phosphorus levels and any safety concerns (maximum 2 mg/kg)			
Outcomes				[Time Frame: up to week 128] Number of subjects for each adverse events Percentage of subjects for each adverse events Effect to body temperature Effect to pulse rate Effect to respiratory rate Effect to blood pressure Effect to 12-Lead Electrocardiogram Effect to Renal Ultrasound Effect to Echocardiogram Serum phosphorus concentration at each test time point 1,25(OH)_2_D at each test time point Alkaline phosphatase at each test time point Urine phosphorus at each test time point Tubular reabsorption of phosphate at each test time point TmP/GFR at each test time point Change from baseline in serum phosphorus Change from baseline in 1,25(OH)_2_D Change from baseline in alkaline phosphatase Change from baseline in urine phosphorus Change from baseline in tubular reabsorption of phosphate Change from baseline in TmP/GFR Improvement in Radiographic Global Impression of Change (RGI-C) global score Change from baseline on Rickets Severity Score (RSS) total score Change from baseline in the Six Minute Walk Test Change in height-for-age z-scores from baseline Serum KRN23 concentration Anti-KRN23 antibody			
Notes				Primary outcome measures were: Number of subjects for each adverse events [Time Frame: up to week 128] Percentage of subjects for each adverse events [Time Frame: up to week 128]			
Martín Ramos, S. et al. (2020) (36)							
ethods				Study design: cohort study Time frame: 2019 to 2020 Follow-up period: one year Excluded: 5			
Participants				Country: Spain Setting: Hospital Patients younger than 18 years of age with XLH genetically confirmed and on treatment with burosumab for more than a year Number: 5 Mean age: 11.00 ± 3.847 (6 to 16) Sex(M/F): 2/3			
Interventions				Burosumab one-year treatment with burosumab, injected subcutaneously at 0.8 mg/kg every 2 weeks			
Outcomes				Serum phosphate Serum AP Serum 1,25(OH)_2_D Serum PTH TRP TmP/GFR Height in cm Skeletal findings Dental abnormalities			
Brener, R. et al. (2022) (35)							
Methods				Study design: cohort study Time frame: 2022 Follow-up period: three years Excluded: 10			
Participants				Country: USA Setting: Pediatric Metabolic Bone Disease Unit in a tertiary medical center. Children with XLH Number: 10 Mean age: 8.80 ± 3.80 (4.3 to 15) Sex(M/F): 4/6			
Interventions				Burosumab SC injections Q2W The dose was adjusted (between 0.8 - 2mg/kg) to achieve a serum phosphorus level at the low end of the normal range for age and for healing the rickets			
Outcomes				Serum phosphate Serum calcium Serum alkaline phosphatase Serum PTH Height, Weight, BMI Rickets severity score Pulp-coronal height ratio Pulp-coronal width ratio			
Notes				Each visit included anthropometric measurements, physical examination, laboratory evaluation and imaging (left hand, wrists, knees and OPT). The routine laboratory evaluation at each time point included serum concentrations of phosphate, calcium, alkaline phosphatase and intact parathyroid hormone.			
Kubota, T. et al. (2023) (39)							
Methods				Study design: cohort study Time frame: 2017 to 2020 Follow-up period: 114.8weeks (range 73.9 – 119.9) Excluded: 20			
Participants				Country: Japan and South Korea Setting: hospital Children with XLH Number: 20 Mean age: 7.40 ± 3.40 (1 to 13) Sex(M/F): 6/14			
Interventions				Burosumab SC injections Q2W A median dose of 17.36 mg (range 7.52 - 51.00 mg) every 2 weeks			
Outcomes				Serum phosphate TmP/GFR Serum 1,25(OH)_2_D Serum iFGF23			
Notes				In the clinical development program of burosumab, self-administration was permitted and monitored in patients with XLH in two open-label, single-arm clinical studies conducted in Japan and South Korea			
Paloian, N. J. et al. (2022) (38)							
Methods				Study design: control study Time frame: 2022 Follow-up period: 3.9 years (1.4 to 16.3) and 24 months Excluded: 12			
Participants				Country: USA Setting: hospital Children with XLH Number: 12 Mean age: Age at XLH diagnosis: 1 (1 to 3) Age at initiation of burosumab: 6 (2 to 18) Sex(M/F): 4/8			
Interventions				Conventional therapy Elemental phosphorus given four times daily and calcitriol given once or twice daily Elemental phosphorus: 20 - 30 mg/kg/day and calcitriol: 20 ng/kg/day Burosumab SC injections Q2W 0.8 mg/kg/dose rounded to the nearest 10 mg in patients <18 years of age and 1 mg/kg/dose rounded to the nearest 10 mg for patients 18 years old and greater			
Outcomes				Serum phosphorus Serum alkaline phosphatase Serum intact PTH Urine FEphosphorus Urine TmP/GFR Urine Ca/Cr			
Levy-Shraga, Y. et al. (2023) (40)							
Methods				Study design: control study Time frame: 2018 to 2021 Follow-up period: 3 years Excluded: 35			
Participants				Country: Israel Setting: Hospital Children with XLH Number: 35 Mean age: 7.5 ± 4.4 (at burosumab initiation) (0.6 to 15.9) Sex(M/F): 18/17			
Interventions				Burosumab SC injections Q2W Initially at a dose of 0.4 to 0.8 mg/kg of body weight, rounded to the nearest 10 mg.			
Outcomes				iFGF23 Phosphorus Calcium Creatinine Alkaline phosphatase iPTH 25-(OH)D 1,25-(OH)_2_ D Calcium/creatinine (urine) TRP TmP/GFR Rickets severity score			
Notes				The dosage was increased stepwise according to laboratory results, consistent with clinical practice guidelines, up to a maximum dose of 2 mg/kg body weight or 90 mg. The study included all the patients who began treatment with burosumab between January 1, 2018, and January 1, 2021.			
Ewert, A. et al. (2023) (31)							
Methods				Study design: control study Time frame: 2022 - 2023 Follow-up period: 12 months Excluded: 93 (Age < 12 y (n = 65); Age ≥ 12 y (n = 28))			
Participants				Country: Germany Setting: Hospital Children and adolescents with XLH Number:93 Mean age:9.6 (5.0 - 12.3) Age < 12y 6.9 (3.4 - 9.7) Age ≥ 12y 13.7 (12.3 - 15.2) Sex(M/F): 34/59			
Interventions				Burosumab SC injections Q2W Initial burosumab dose of 0.4 mg/kg body weight given every 2 weeks			
Outcomes				Phosphate TmP/GFR TRP ALP PTH 25-(OH)D 1,25-(OH)_2_ D U _Ca/Crea_			
Notes				Reduced fasting, age-related serum Pi levels, after a washout period of at least 7 days in patients on conventional treatment. Titration of burosumab dose in increments of 0.4 mg/kg body weight to raise fasting serum Pi levels within the lower end of the normal age reference range, with a maximum dosage of 2.0 mg/kg body weight (maximum dose 90 mg). Discontinuation of burosumab if fasting serum Pi level were above the upper normal limit (ULN). Prior to the study period, 33 (94%) patients received conventional therapy, namely oral phosphate supplement and alfacalcidol.			

### 3.3 Risk of bias in individual studies

The Newcastle–Ottawa scale was applied to determine included study quality (**Tables 2**, **3**). Ten included studies were categorized as either randomized-control or cohort studies, and different evaluation forms were employed to assess them accordingly. The eight cohort studies scored 7-8 points, denoting high quality (31, 35–37, 39–42) (**Table 2**), while the two randomized-control studies achieved full marks, designating them as high-quality articles (32, 38) (**Table 3**).

Table 2Quality assessment of the included studies based on the Newcastle‐Ottawa scale (cohort studies).

**Table:** 

	Selection^a^				Comparability^b^	Exposure^c^			
Author	Representativeness of the exposed cohort^e^	Selection of the non-exposed cohort^f^	Ascertainment of exposure^g^	Demonstration that outcome of interest was not present at start of study^h^	Comparability of cases and controls on the basis of the design or analysis^i^	Assessment of outcome^j^	Was follow-up long enough for outcomes to occur^k^	Adequacy of follow up of cohorts^l^	Score^d^
Linglart, A. et al. (2019) (41)	✯	✯	✯	_	✯	✯	✯	✯	7
Namba, N. et al. (2022) (37)	✯	✯	✯	_	✯✯	✯	✯	✯	8
Whyte, M. P. et al. (2018) (42)	✯	✯	✯	_	✯	✯	✯	✯	7
Martín Ramos, S. et al. (2020) (36)	✯	✯	✯✯	_	✯	✯	✯	✯	8
Brener, R. et al. (2022) (35)	✯	✯	✯✯	_	✯	✯	✯	✯	8
Kubota, T. et al. (2023) (39)	✯	✯	✯	_	✯	✯	✯	✯	7
Ewert, A. et al. (2023) (31)	✯	✯	✯	_	✯✯	✯	✯	✯	8
Levy-Shraga, Y. et al. (2023) (40)	✯	✯	✯	_	✯	✯	✯	✯	7

✯:one point

aA maximum of one point for each item. ^b^A maximum of two points for each item. ^c^A maximum of one point for each item. ^d^A maximum of nine points. ^e^(a)truly representative of the average XLH in the community✯, (b) somewhat representative of the average XLHin the community✯,(c) selected group of users eg nurses, volunteers,(d)no description of the derivation of the cohort. ^f^(a) drawn from the same community as the exposed cohort✯, (b)drawn from a different source,(c)no description of the derivation of the non-exposed cohort. ^g^(a) secure record (eg surgical records) ✯,(b) structured interview ✯, (c)written self-report,(d)no description. ^h^(a) yes✯, (b) no. ^i^(a) the exposures of interest (Burosumab and active control) were adjusted for one confounder (sex or age) ✯, (b) the exposures of interest (burosumab and active controll) were adjusted for two or more confounders (sex or age and treatment compliance or severity of illness) ✯✯, c) no description. ^j^(a) independent blind assessment✯, (b)record linkage✯, (c) self-report, (d)no description. ^k^(a) yes (select an adequate follow up period for outcome of interest)✯, (b) no. ^l^(a) complete follow up - all subjects accounted for ✯, (b) subjects lost to follow up unlikely to introduce bias - small number lost - > 80% follow up, or description provided of those lost)✯, (c) follow up rate < 80% and no description of those lost, (d)no statement.

**Table 3:** Quality assessment of the included studies based on the Newcastle‐Ottawa scale (randomized control studies).

	Selection^a^				Comparability^b^	Exposure^c^			
Author	Is the case definition adequate?^e^	Representativeness of the cases^f^	Selection of Controls^g^	Definition of Controls^h^	Comparability of cases and controls on the basis of the design or analysis^i^	Ascertainment of exposure^j^	Same method of ascertainment for cases and controls^k^	Non‐ Response rate^l^	Score^d^
Imel, E. A. et al. (2019) (32)	✯	✯	✯	_	✯✯	✯	✯	✯	8
Paloian, N. J. et al. (2022) (38)	✯	✯	✯	_	✯✯	✯	✯	✯	8

✯:one point

aA maximum of one point for each item. ^b^A maximum of two points for each item. ^c^A maximum of one point for each item. ^d^A maximum of nine points. ^e^(a) yes, with independent validation ✯, (b) yes, for example, record linkage or based on self‐reports, (c) no description. ^f^(a) consecutive or obviously representative series of cases ✯, (b) potential for selection biases or not stated. ^g^(a) community controls ✯,(b) hospital controls, (c) no description. ^h^(a) no history of disease (endpoint) ✯, (b) no description of source. ^i^(a) the exposures of interest (burosumab and active control) were adjusted for one confounder (sex or age) ✯, (b) the exposures of interest (burosumab and active controll) were adjusted for two or more confounders (sex or age and treatment compliance or severity of illness) ✯✯, c) no description. ^j^(a) secure record (e.g., surgical records) ✯, (b)structured interview where blind to case/control status ✯, (c) interview not blinded to case/control status, (d) written self‐report or medical record only, (e) no description. ^k^(a) yes ✯, (b) no. ^l^(a) same rate for both groups ✯, (b) non‐respondents described, (c) rate different and no designation.

### 3.4 Meta-analysis for the effects of burosumab

For the meta-analysis of burosumab treatment outcomes, forest plots were employed to comprehensively assess overall effects, including 1,25-(OH)_2_D, serum phosphorus, TmP/GFR, RSS, ALP, 6MWT, and Height Z score.

#### 3.4.1 1,25-(OH)_2_D

**Figure 2:**
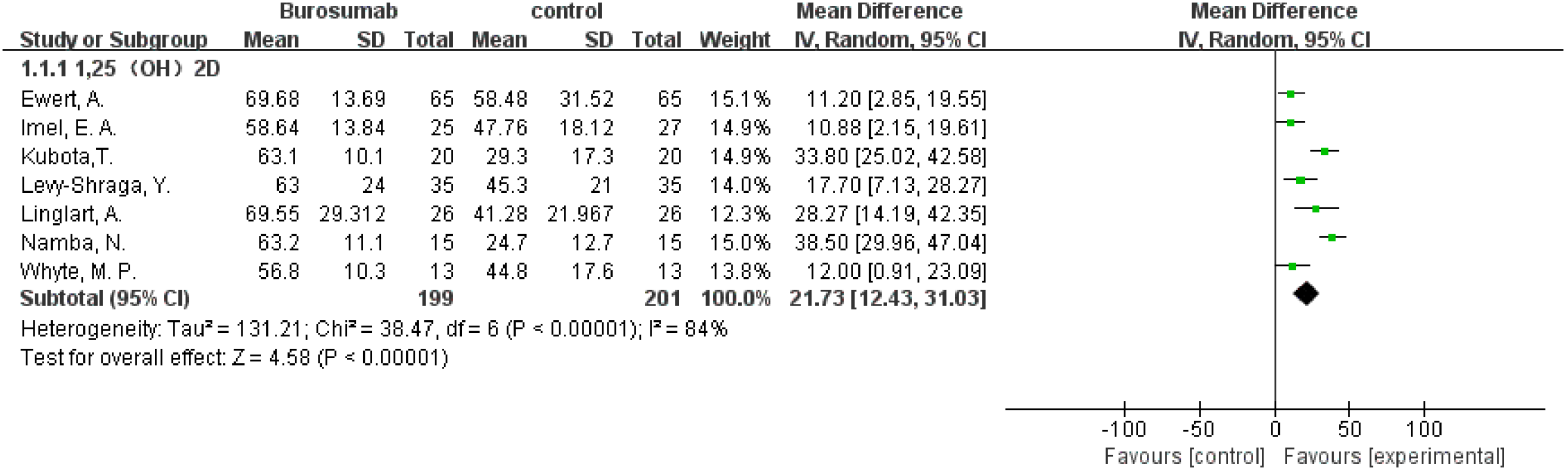
Meta-meta-analysis of the effects of burosumab on serum 1,25-dihydroxyvitamin D (1,25-(OH)_2_D). The bottom row describes a combined overall effect of treatment which random-effects models were used to estimate.

**Figure 3:**
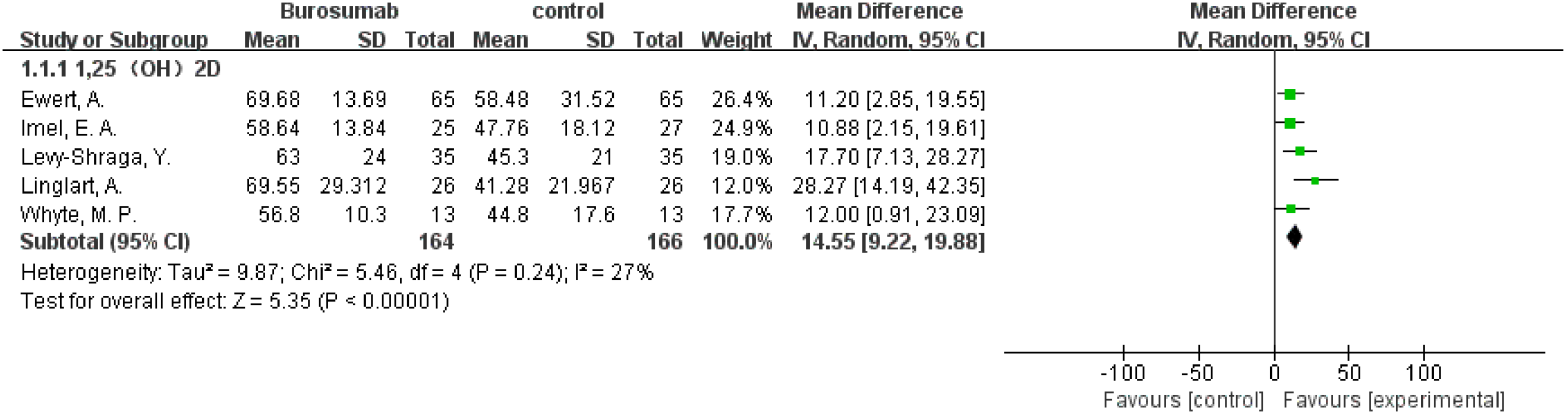
Sensitivity analysis of the effects of burosumab on serum 1,25-dihydroxyvitamin D (1,25-(OH)_2_D). The bottom row describes a combined overall effect of treatment, which is similar to previous result.

Seven studies with a total of 400 patients were included in the analysis of changes in 1,25-(OH)_2_D before and after therapy (31, 32, 37, 39–42) (**Figure 2**). A significantly greater increase in 1,25-(OH)_2_D levels in the burosumab group compared to the control group (SMD 21.73, 95% CI 12.43-31.03, P <0.00001), albeit with considerable heterogeneity (I^2^ = 84%). Sensitivity analysis pinpointed two studies, Kubota, T. et al. and Namba, N. et al. (37, 39) as major heterogeneity sources due to their low baseline values. Excluding these studies did not alter the overall conclusion, reinforcing burosumab’s effectiveness in addressing vitamin D metabolism in pediatric XLH patients (**Figure 3**).

#### 3.4.2 Serum phosphorus

**Figure 4:**
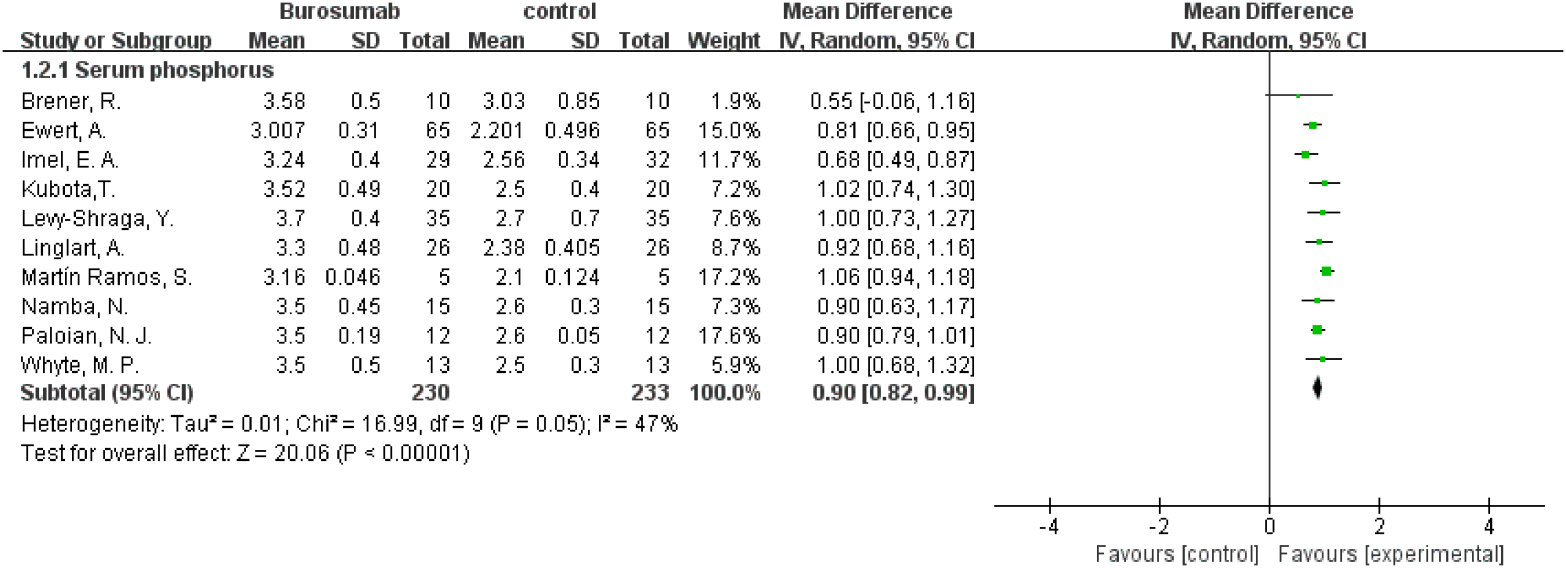
Meta-meta-analysis of the effects of burosumab on serum phosphorus. The bottom row describes a combined overall effect of treatment which random-effects models were used to estimate.

This outcome was analyzed using data from ten studies (31, 32, 35–42) (**Figure 4**). The analysis of these ten publications collectively demonstrated a notable increase in serum phosphorus levels in the burosumab group compared to controls (SMD 0.9, 95% CI 0.82-0.99, P<0.0001), with moderate heterogeneity (I^2^ = 47%). The analysis robustly demonstrates burosumab’s role in correcting hypophosphatemia in XLH children.

#### 3.4.3 TmP/GFR

**Figure 5:**
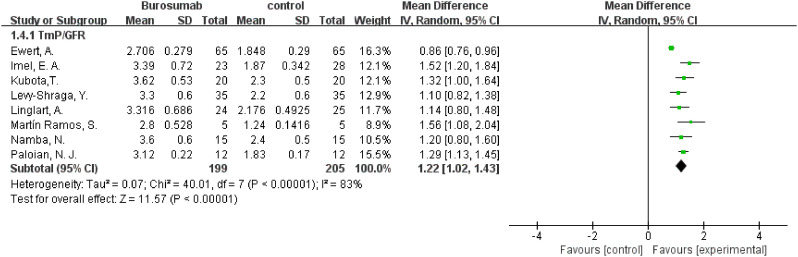
Meta-meta-analysis of the effects of burosumab on renal tubular maximum reabsorption rate of phosphate to glomerular filtration rate (TmP/GFR). The bottom row describes a combined overall effect of treatment which random-effects models were used to estimate.

**Figure 6:**
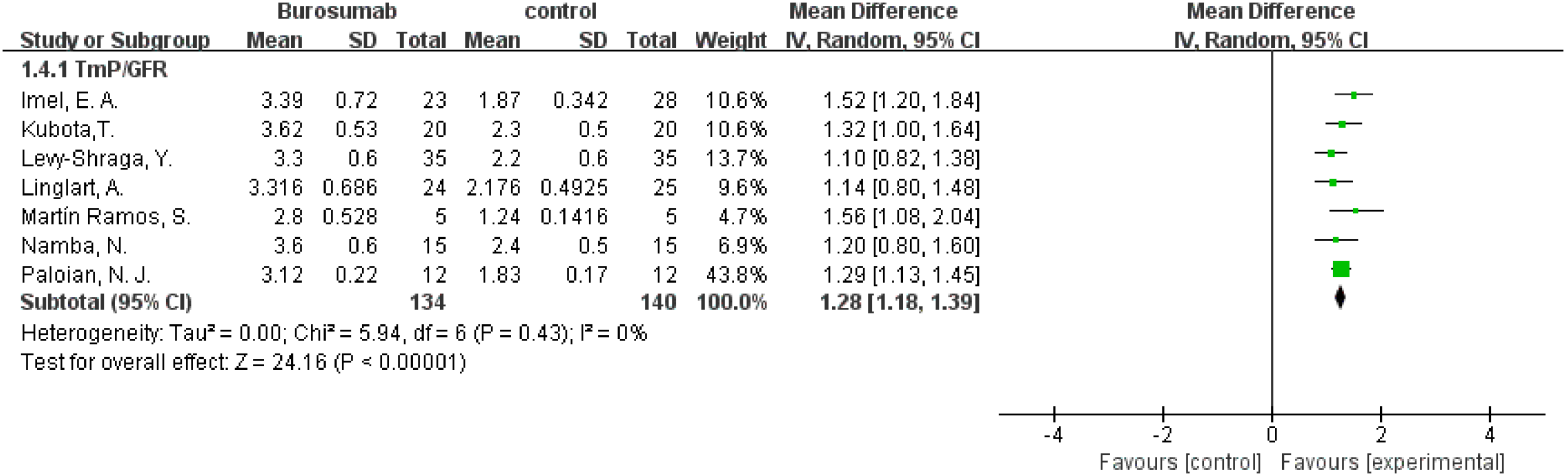
Sensitivity analysis of the effects of burosumab on renal tubular maximum reabsorption rate of phosphate to glomerular filtration rate (TmP/GFR). The bottom row describes a combined overall effect of treatment, which is similar to previous result.

To evaluate changes in TmP/GFR before and after therapy, data from eight studies were used in this analysis (31, 32, 36–41) (**Figure 5**). The analysis demonstrated a significant increase in TmP/GFR in the burosumab group (SMD 1.22, 95% CI 1.02-1.43, P<0.00001). The I^2^ statistic was 83%, showing a significant heterogeneity. Sensitivity analysis showed that the heterogeneity came from Ewert, A. et al. study (31), which had a lower Mean Difference (MD) (**Figure 6**). Exclusion of this study leaded to consistent conclusion. The significant improvement in TmP/GFR ratios with burosumab treatment, even after adjusting for heterogeneity, underscores its effectiveness in enhancing renal phosphate reabsorption.

#### 3.4.4 RSS

**Figure 7:**
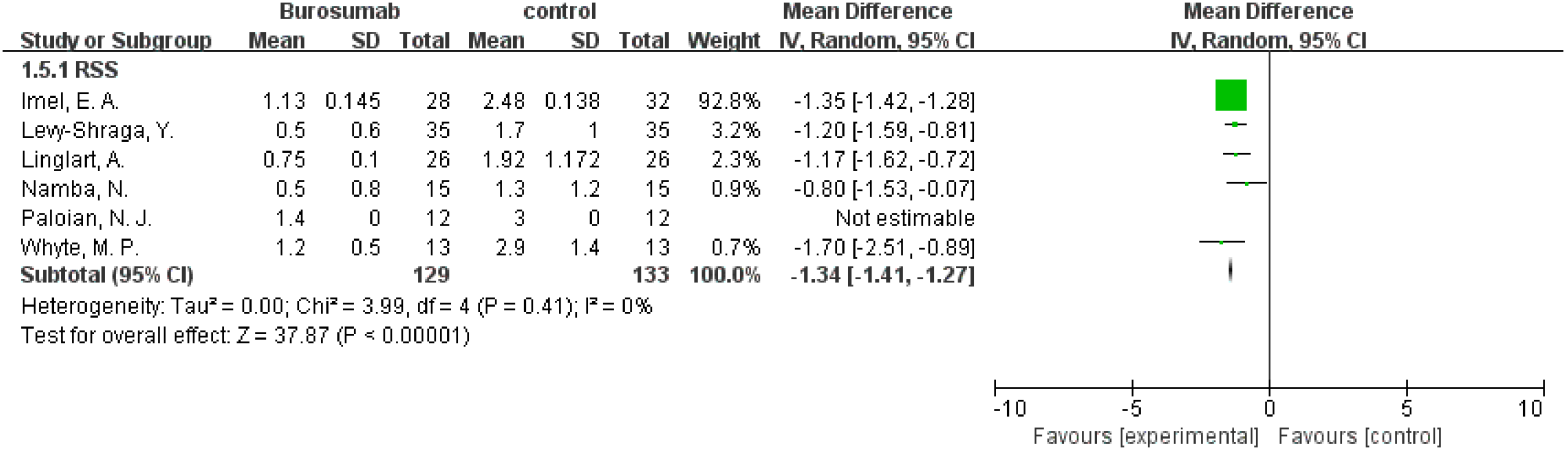
Meta-meta-analysis of the effects of burosumab on rickets severity score (RSS). The bottom row describes a combined overall effect of treatment which random-effects models were used to estimate.

Six studies contributed to RSS analysis (32, 37, 38, 40–42) (**Figure 7**). Burosumab group showed a significant reduction in RSS compared to controls (95% CI 1.41-1.27, P<0.00001), without significant heterogeneity (I^2^ = 0%). The reduction in RSS scores in the burosumab group strongly suggests that burosumab is effective in improving radiographic outcomes for XLH patients.

#### 3.4.5 ALP

**Figure 8:**
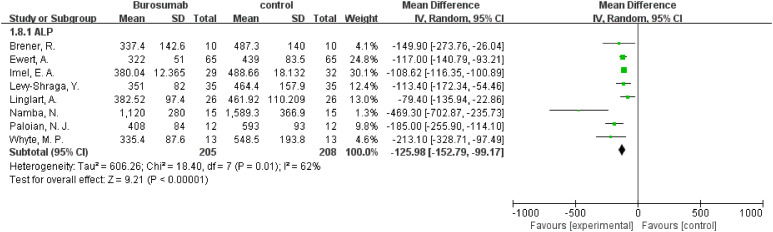
Meta-meta-analysis of the effects of burosumab on serum alkaline phosphatase (ALP). The bottom row describes a combined overall effect of treatment which random-effects models were used to estimate.

**Figure 9:**
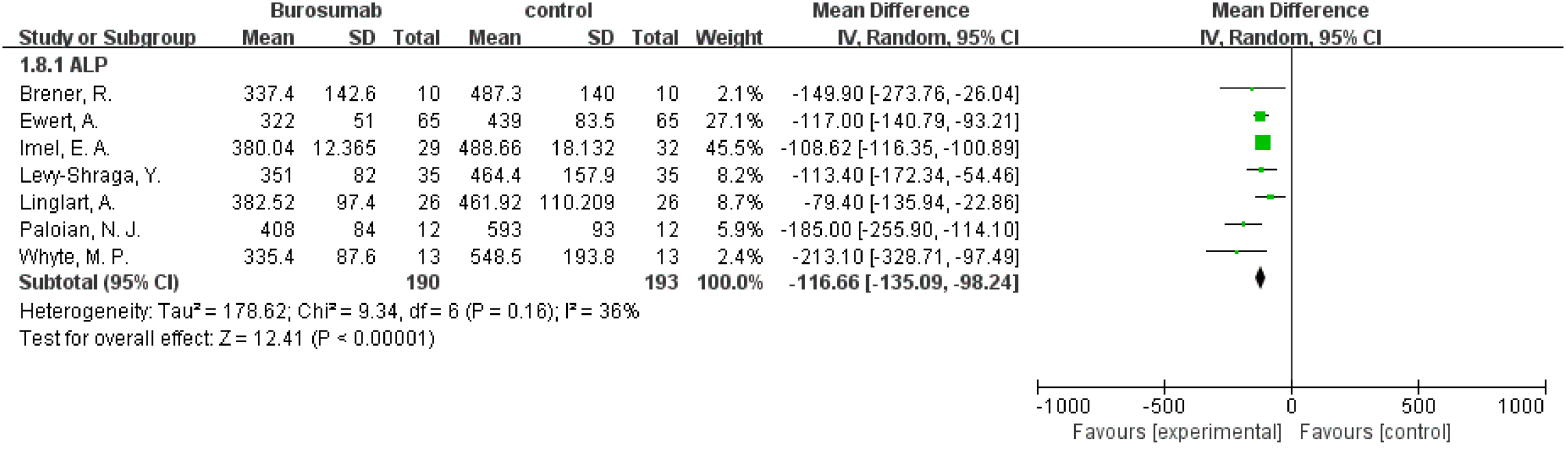
Sensitivity analysis of the effects of burosumab on serum alkaline phosphatase (ALP). The bottom row describes a combined overall effect of treatment, which is similar to previous result.

Eight studies were analyzed for ALP parameter, indicating a significant reduction in ALP levels in the burosumab group (31, 32, 35, 37, 38, 40–42) (**Figure 8**) (SMD -125.98, 95% CI −152.79-−99.17, P<0.00001). The I^2^ statistic was 62%, showing a significant heterogeneity. Sensitivity analysis showed that the heterogeneity came from Namba, N. et al. (37). study, which exhibited a significantly higher ALP value compared to other studies (**Figure 9**), and heterogeneity was resolved by excluding this study. The substantial decrease in ALP levels in patients treated with burosumab indicates its efficacy in normalizing bone turnover markers in XLH.

#### 3.4.6 6MWT

**Figure 10:**
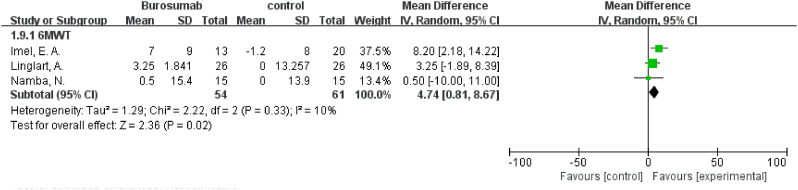
Meta-meta-analysis of the effects of burosumab on 6-minute walking test (6MWT). The bottom row describes a combined overall effect of treatment which random-effects models were used to estimate.

The assessment of changes in the 6MWT included three studies (32, 37, 41), showing the burosumab group’s 6MWT was 4.74 longer than the control group (95% CI 0.81-8.67, P=0.02), with no significant heterogeneity (I^2^ = 10%) (**Figure 10**). The improvement in the 6MWT distances in the burosumab group points to enhanced physical functioning and endurance in treated patients.

#### 3.4.7 Height Z score

**Figure 11:**
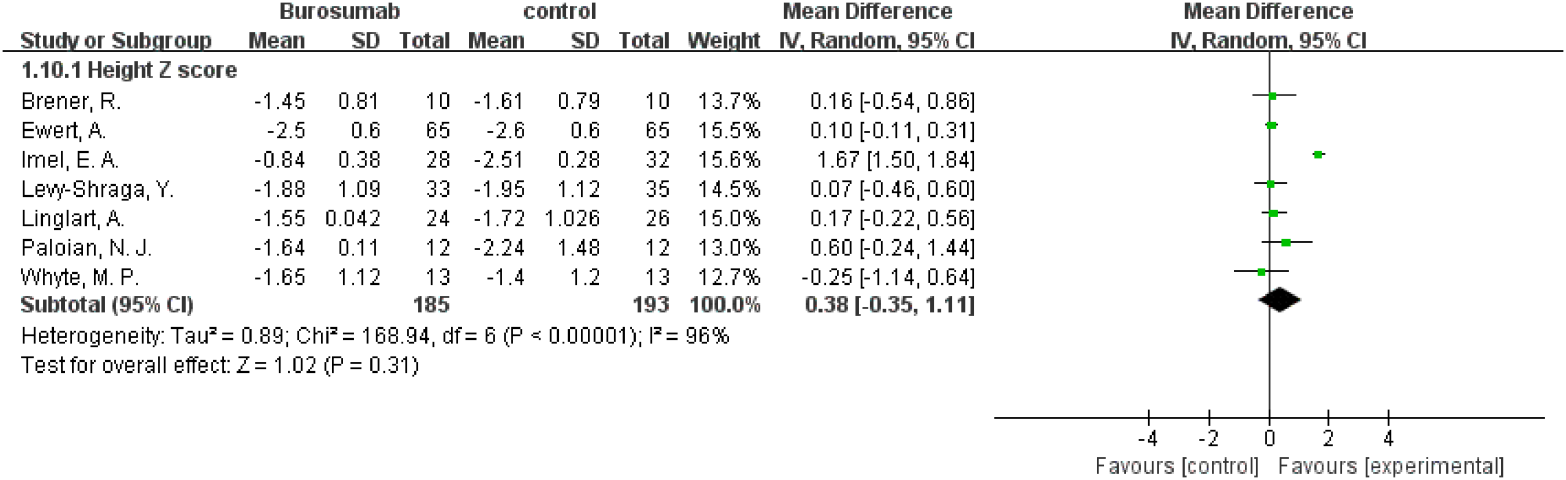
Meta-meta-analysis of the effects of burosumab on Hight Z score. The bottom row describes a combined overall effect of treatment which random-effects models were used to estimate.

Changes in the Height Z score before and after treatment were assessed by seven studies (31, 32, 35, 38, 40–42) (**Figure 11**). The analysis showed no significant change in Height Z score (SMD 0.38, 95% CI −0.35-1.1, P=0.31) with high heterogeneity (I²=96%), rendering the results inconclusive. The inconclusive results regarding changes in Height Z scores, accompanied by significant heterogeneity, suggest the need for further research to fully understand burosumab’s impact on growth in XLH patients.

#### 3.4.8 Subgroup analysis

**Figure 12:**
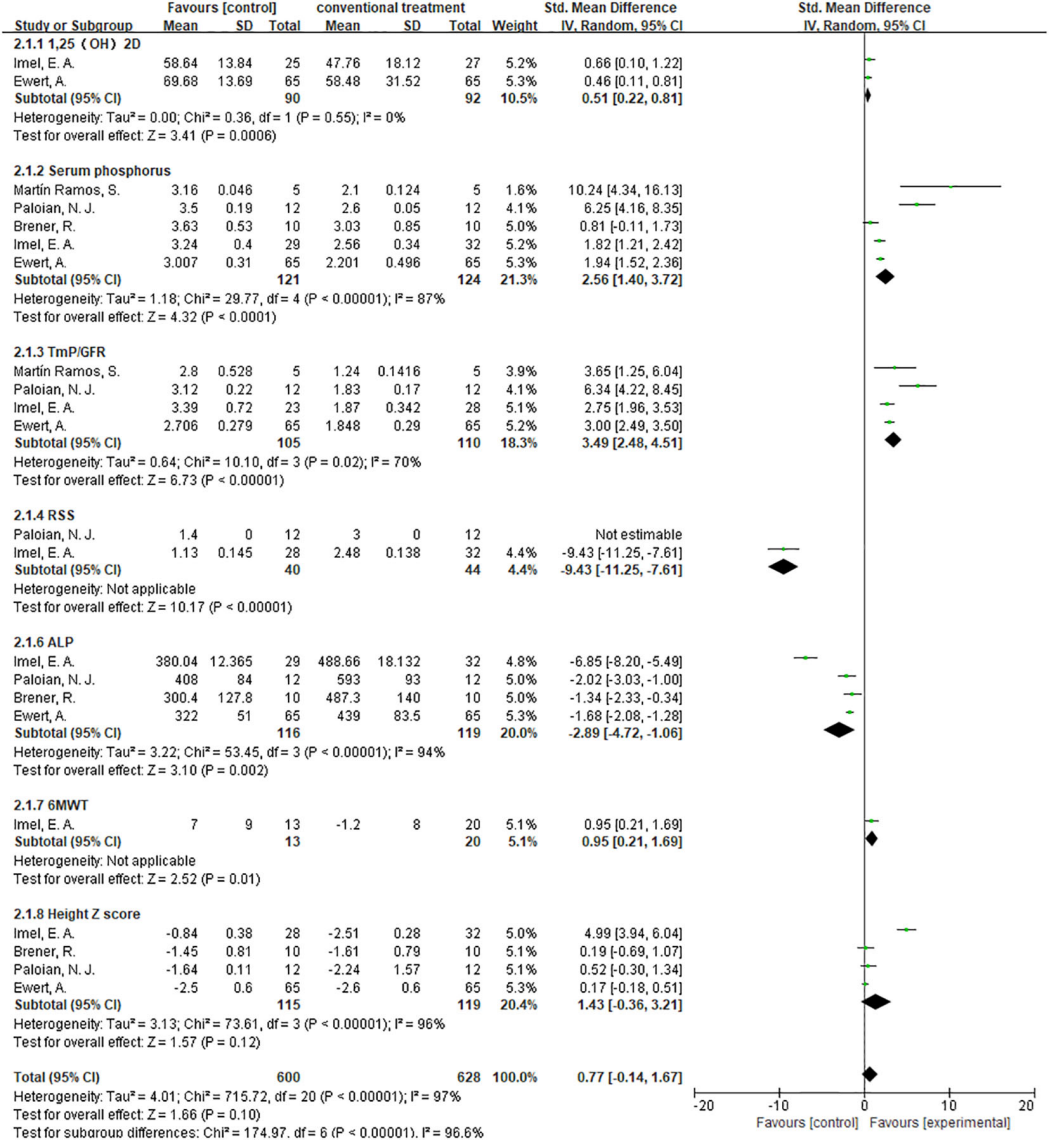
Meta-meta-analysis of the effect between burosumab and conventional therapy. The indexes include 1,25-dihydroxyvitamin D (1,25-(OH)_2_D), serum phosphorus, renal tubular maximum reabsorption rate of phosphate to glomerular filtration rate (TmP/GFR), rickets severity score (RSS), serum alkaline phosphatase (ALP), 6-minute walking test (6MWT) and Hight Z score. The bottom row describes a combined overall effect of treatment which random-effects models were used to estimate.

Comparing burosumab to traditional phosphate and active vitamin D supplements in treating pediatric XLH patients is crucial due to burosumab’s targeted mechanism of inhibiting FGF23, potentially offering more direct correction of the underlying phosphate wasting. This comparison is essential to assess burosumab’s effectiveness in improving bone health, its convenience with less frequent dosing enhancing patient adherence, and its side effect profile relative to conventional treatments that often come with gastrointestinal issues and risk of secondary hyperparathyroidism. Moreover, understanding the long-term impacts on growth, skeletal abnormalities, and cost-effectiveness given burosumab’s anticipated higher costs, is vital for providing evidence-based recommendations for managing XLH. To this end, we have performed a subgroup analysis of trials that contrast patients treated with burosumab against those receiving conventional therapy. The outcomes of this analysis could offer crucial insights for patients contemplating their future treatment choices (**Figure 12**).

##### 3.4.8.1 1,25-(OH)_2_D

The subgroup analysis of three studies (31, 32, 37) demonstrated a more significant increase in 1,25-(OH)_2_D levels in the burosumab group compared to traditional therapies, with a SMD of 1.27 (95% CI 0.17-2.38, P=0.02). This significant improvement suggests burosumab’s superior efficacy in correcting the aberrant vitamin D metabolism associated with XLH.

##### 3.4.8.2 Serum Phosphorus

The analysis, incorporating data from six studies (31, 32, 35–38), showed a pronounced improvement in serum phosphorus levels in the burosumab-treated group, with an SMD of 2.43 (95% CI 1.49-3.37, P<0.00001). This finding highlights burosumab’s potent effect on ameliorating hypophosphatemia, a hallmark of XLH.

##### 3.4.8.3 TmP/GFR

Combining results from both randomized-control (32, 38) and cohort controls (31, 36, 37) revealed a substantial increase in TmP/GFR in the burosumab group, with an SMD of 3.14 (95% CI 2.29-3.99, P<0.00001). This indicates burosumab’s effectiveness in enhancing renal phosphate reabsorption, further supporting its therapeutic advantage in XLH management.

##### 3.4.8.4 ALP Levels

The subgroup analysis of five studies (31, 32, 35, 37, 38) indicated a significant reduction in ALP levels for patients treated with burosumab, with an SMD of -2.43 (95% CI -3.93 to -1.19, P=0.0002). This outcome reflects burosumab’s capacity to normalize bone turnover markers, suggesting improved bone metabolism in treated patients.

##### 3.4.8.5 Height Z Score

The analysis of changes in Height Z score yielded inconclusive results. The SMD was 1.43 (95% CI -0.36 to 3.21, P=0.12), with high heterogeneity. This outcome signifies the complexity of assessing burosumab’s impact on growth and necessitates further longitudinal studies to elucidate this aspect.

Given that RSS and 6MWT each have only a single valid study comparing burosumab to conventional therapy, subgroup analysis was not conducted for these two metrics.

The subgroup analysis elucidates burosumab’s efficacy over conventional therapies across several key parameters, underscoring its potential to offer a more targeted and effective treatment for XLH. However, the variability in outcomes, especially regarding growth (Height Z score), underscores the necessity for ongoing research to fully understand burosumab’s long-term benefits and implications in pediatric XLH treatment.

## 4 Discussion

Children with X-linked hypophosphatemia (XLH) often face significant challenges due to the debilitating effects of rickets and osteomalacia. These conditions not only compromise their physical well-being but also impact their quality of lives. While conventional treatments have included vitamin D metabolites and phosphate supplements, the emergence of burosumab has introduced a promising alternative in ameliorating the negative effects of XLH.

This meta-analysis comprehensively evaluated the efficacy of burosumab in the treatment of pediatric XLH patients. Ten high-quality clinical studies were systematically analyzed, revealing significant improvements in key parameters such as 1,25-(OH)_2_D levels, serum phosphorus, TmP/GFR, RSS, ALP levels, and 6MWT performance (31, 32, 35–42). Despite limitations in some individual studies, the findings provide compelling evidence supporting burosumab as an effective treatment for XLH, offering significant benefits in various aspects of patient health and well-being.

The subgroup comparison between burosumab treatment and conventional therapy within the context of pediatric XLH patients reveals crucial insights into the effectiveness of these two approaches (31, 32, 35–38). As elucidated in this meta-analysis, the comparison consistently indicates that burosumab is superior to conventional treatments in increasing serum phosphorus levels and active vitamin D levels (31, 32, 37), enhancing renal phosphate reabsorption (31, 32, 36–38), and decreasing ALP levels (31, 32, 35, 37, 38), underscoring its potential to offer XLH patients a more effective and comprehensive treatment solution. However, limited data on RSS and 6MWT performance mean that further studies are needed to conclusively compare these treatments. This finding is crucial for clinicians and patients in deciding the most effective therapeutic approach for managing pediatric XLH.

One notable aspect illuminated by this analysis is the multifaceted impact of burosumab. Beyond improvement in biochemical markers, the drug demonstrates tangible benefits in the form of enhanced mobility, reduced bone deformities, and overall quality of life. This emphasizes the importance of evaluating XLH treatments beyond traditional biochemical markers, focusing also on functional and quality-of-life outcomes. Burosumab’s influence on RSS (35, 37, 38, 40–42) and 6MWT (32, 37, 41) signifies this effective approach, addressing not only biochemical imbalances but also the functional limitations experienced by patients. This perspective aligns with the patient-centered care paradigm, emphasizing treatments that address both physiological and real-world challenges faced by pediatric XLH patients.

The XLH patients before burosumab treatment is generally poor due to the numerous physical and psychological challenges associated with the disorder, including chronic pain, skeletal deformities, and impaired mobility. Many patients require corrective orthopedic surgeries due to severe bone deformities, which add to the physical and emotional burden. Despite the promising results of burosumab therapy in treating XLH in biochemical markers, there is currently limited QOL data available on its use. Two reports indicate that burosumab has been effective in reducing bone pain and correcting skeletal deformities, significantly improving mobility and physical comfort, as well as enhancing happiness and life satisfaction for patients (28, 29). However, comprehensive data on how burosumab impacts patients’ overall well-being, daily functioning, and social interactions are still needed. This lack of extensive QOL data highlights the need for further research to fully understand the therapy’s benefits and drawbacks from the patients’ perspectives. Understanding the effects of burosumab on QOL is crucial for optimizing treatment plans and ensuring that the therapy not only addresses the clinical manifestations of XLH but also enhances the overall life satisfaction and daily functioning of those affected.

In addition to QOL concerns, several unknown clinical questions regarding its short-term effects need to be addressed through ongoing research and clinical practice. Immediate side effects, such as injection site reactions and hypersensitivity, require more comprehensive data across different age groups and patient populations. Additionally, the optimal dosage and administration schedule for various patient demographics need further refinement to ensure effective and safe treatment. Monitoring short-term biochemical responses, including changes in serum phosphate and ALP levels, is crucial for predicting treatment outcomes and adjusting therapy. While phosphate levels have been consistently used to monitor burosumab therapy and have shown significant improvement in this meta-analysis, recent findings suggest that ALP is a more stable and reliable marker. Many patients experience sustained growth and ALP normalization on burosumab treatment without achieving normal plasma phosphate concentration (31, 43). ALP provides a consistent biochemical indicator of bone activity, which allows for a more accurate and comprehensive assessment of the therapy’s effectiveness in children. Evaluating the immediate impact on bone health markers, such as bone density, growth rates in children, and reductions in bone pain and fractures, is also essential. Assessing the short-term impact on patients’ quality of life, including pain reduction, mobility, and daily functioning, provides a holistic view of its benefits. Understanding patient responses during the acute phase of burosumab treatment is important for setting patient expectations and managing care effectively. Addressing these questions through rigorous clinical trials will be crucial to fully understanding the short-term effects of burosumab therapy and optimizing its use in managing XLH.

Additionally, the meta-analysis raises critical questions about the long-term safety and efficacy of burosumab. While the analyzed studies provide encouraging short-to-medium-term outcomes, the long-term impacts remain uncertain. Longitudinal studies tracking patients over several years are necessary to ascertain the sustainability of burosumab’s benefits. Furthermore, the potential for adverse effects in the context of prolonged monoclonal antibody therapy warrants careful consideration, demanding a comprehensive risk-benefit analysis.

Moreover, the findings of this meta-analysis accentuate the importance of individualized treatment approaches (41). XLH is a heterogeneous disorder, exhibiting significant variability in its clinical manifestations. Personalized approaches tailoring burosumab therapy to the specific needs of each patient is crucial. Personalized approaches taking into account individual factors such as age, disease severity, and comorbid conditions, could enhance therapeutic outcomes and minimize risks. There was one study that assessed the variance in burosumab treatment efficacy among children and adolescents. The findings indicated that there was no discernible difference in burosumab’s impact on both age groups when indices like ALP levels, RSS value, serum phosphate, and TmP/GFR returned to normal (31). However, compared to younger children, adolescents require a lower unit dosage of burosumab.

There is evidence of sexual dimorphism in the severity of XLH, with males often exhibiting more severe symptoms than females. This difference is particularly noticeable in skeletal and dental impairments, aligning with the notion that males tend to have more severe mineralization defects (5, 30). The severity in males is thought to be influenced by variations in sex hormones, physical activity, and other factors (44). Burosumab has shown effectiveness in improving biochemical markers and physical symptoms in both males and females with XLH (37, 40). Similar improvements in serum phosphate levels, active vitamin D levels, renal phosphate reabsorption, as well as physical symptoms were observed in both males and females. More detailed studies focusing on long-term outcomes and direct comparisons between males and females are needed to fully understand the nuances of burosumab’s effectiveness across different patient groups. Such research is crucial to optimize treatment strategies and ensure that all patients, regardless of sex, achieve the best possible outcomes.

According to clinical practice guidelines for pediatric X-linked hypophosphatemia in the era of burosumab, it is widely endorsed an initial dosage of 0.8 mg/kg of body weight (which is changed to 0.4 mg/kg in Europe), rounded to the nearest 10 mg (maximum dose 90 mg), administered subcutaneously every 2 weeks (45). Once burosumab therapy commences, fasting serum phosphate levels should be monitored every 4 weeks for the initial 3 months. Dose adjustments are made based on changes in serum phosphate levels and other parameters, aiming to maintain them within the normal range for the child’s age. For instance, if fasting serum phosphate falls below the normal range on two consecutive occasions (4 weeks apart), the dose should be increased. Conversely, if the level exceeds the normal range, the dose should be withheld and later resumed at a reduced level once it falls below the normal range. In relevant articles analyzed, Brener et al. tailored the dose (ranging from 0.8-2 mg/kg) to attain a serum phosphorus level at the lower limit of the normal range for age and facilitate rickets healing (35). Ewert et al. observed significant variations in the final weight-based burosumab doses among children aged 1 to 12, with an interquartile range of 0.72 to 1.41 mg/kg, yet failed to establish a notable correlation between these doses and other parameters (31). Notably, our findings indicate that dosage adjustments are common in most studies, and even the exhaustion of the maximum recommended dosage may not always lead to normalization of serum phosphorus level and TmP/GFR, implying that dosage variations stem from diverse patient conditions.

There is evidence that burosumab is also effective in adult XLH patients. In a double-blind, placebo-controlled phase 3 trial, adults with XLH received subcutaneous injections of 1:1 burosumab 1 mg/kg (n = 68) or placebo (n = 66) every 4 weeks. The trial demonstrated that burosumab increased renal phosphate reabsorption and normalized serum phosphate levels throughout the dosing interval in symptomatic adults with XLH by binding to excess circulating FGF23 (46). 1,25(OH)_2_D and TmP/GFR concentrations were higher in the burosumab group compared to the placebo group. Improvements in phosphate metabolism were accompanied by significant reductions in stiffness, increased body function, and decreased pain, which may be expected to improve mineralization and restore normal bone physiology. Additionally, another trial of burosumab treatment in adults demonstrated that burosumab can bring about a possible positive remodeling balance in which the serum markers of bone turnover were improved (47). The results in the 6MWT were used to evaluate exploratory efficacy endpoints for mobility and they were returned to normal levels after burosumab treatment. It also suggested that the efficacy of burosumab can lasts for more than 3 years on average, with no evidence of diminished or impaired clinical response after reintroduction of the drug after treatment interruption.

The cost-benefit balance between burosumab and conventional therapy is an important consideration in real-life practice. Burosumab therapy is more than 100 times higher in cost than conventional therapy, with an annual expense of approximately $160,000 per patient for children and $200,000 per patient for adults (49). The decision to use burosumab over conventional therapy involves evaluating these significant cost differences against the potential for better long-term outcomes and reduced complications with burosumab.

In conclusion, burosumab represents a significant advancement in treating pediatric XLH, yet the treatment landscape for this rare disorder is still evolving. Future research should focus on unraveling the molecular mechanisms of XLH and the specific action mode of burosumab. Additionally, exploring combinational therapies integrating burosumab with other targeted treatments could represent the next frontier in XLH management. A multidisciplinary approach, integrating genetics, molecular biology, and clinical expertise, is essential to further improve outcomes for individuals with XLH, aiming for minimal life impact from the disorder.
